# The triglyceride paradox in the mortality of coronary artery disease

**DOI:** 10.1186/s12944-019-0972-0

**Published:** 2019-01-22

**Authors:** Tian-li Xia, Yi-ming Li, Fang-yang Huang, Hua Chai, Bao-tao Huang, Qiao Li, Zhen-gang Zhao, Yan-biao Liao, Zhi-liang Zuo, Yong Peng, Mao Chen, De-jia Huang

**Affiliations:** 1 Department of Cardiology, West China Hospital, Sichuan University, 37 Guoxue Street, Chengdu, 610041 People’s Republic of China; 2 West China School of Medicine, Sichuan University, Chengdu, China

**Keywords:** Prognosis, Hypertriglyceridemia, Paradox

## Abstract

**Background:**

The role of triglyceride (TG) in secondary prevention of patients with coronary artery disease (CAD) was debated. In the present study, we assessed the association between admission TG levels and long-term mortality risk in CAD patients.

**Methods:**

A retrospective analysis was conducted from a single registered database. 3061 consecutive patients with CAD confirmed by coronary angiography were enrolled and were grouped into 3 categories by the tertiles of admission serum TG levels. The primary end point in this study was all-cause mortality and the secondary end point was cardiovascular mortality.

**Results:**

The mean follow-up time was 26.9 ± 13.6 months and death events occurred in 258 cases and cardiovascular death events occurred in 146 cases. Cumulative survival curves indicated that the risk of all-cause death decreased with increasing TG level (Tertile 1 vs. Tertile 2 vs. Tertile 3 = 10.3% vs. 8.6% vs. 6.3%, log rank test for overall *p* = 0.001). Cox regression analysis showed an independent correlation between TG level and risk of all-cause mortality [hazard ratio (HR) 0.71, 95% confidence interval (CI) 0.58–0.86] and cardiovascular mortality (HR 0.67, 95% CI 0.51–0.89) in total patients with CAD. Subgroup analysis found the similar results in patients with acute coronary syndrome and acute myocardial infarction.

**Conclusions:**

This study found an inverse association between TG levels and mortality risk in CAD patients, which suggests that the “TG paradox” may exist in CAD patients.

**Trial registration:**

ChiCTR, ChiCTR-OOC-17010433. Registered 17 February 2017 - Retrospectively registered.

## Introduction

Hypertriglyceridemia (HTG) is considered a risk factor for atherosclerotic cardiovascular disease (ASCVD) [1]. Epidemiological studies showed that coronary artery disease (CAD) patients have higher triglyceride (TG) levels than the general population [2, 3]. Additionally, cohort studies showed that HTG can increase the risk of suffering from CAD [4–6]. However, the association between TG and ASCVD risk has been controversial. A series of studies suggested that there is no independent association between HTG and ASCVD risk after multifactor adjustment for cholesterol (TC), low-density lipoprotein cholesterol (LDL-C), and high-density lipoprotein cholesterol (HDL-C) [7]. The role of HTG was also debated in secondary prevention studies of patients who suffer from CAD. Some studies suggested that HTG increases the mortality risk in patients with CAD [8, 9]; however, other studies suggested that there is no association between the two factors [10]. Several recent studies found an inverse association between admission TG level and mortality risk in stroke patients; these studies then proposed the concept of the “TG paradox” [11–14]. As one of ASCVD, CAD has the similar pathophysiological mechanism. Whether a similar TG paradox exists in CAD patients remains unclear.

In the present study, we assessed the association between admission TG levels and long-term mortality risk in CAD patients through analyzing a single-center cohort of 3061 consecutively enrolled patients with CAD.

## Methods

### Study population

The data source for this investigation was the West China Hospital CAD database. This single center database prospectively includes all the CAD or high risk patients undergoing angiography in West China Hospital affiliated to Sichuan University. For this analysis, we enrolled consecutive patients with CAD from January 2009 to September 2012 of the database. Patients with CAD were eligible for inclusion if they were restricted to participants with angiographic evidence of ≥50% stenosis in ≥1 coronary vessels. The criteria of acute myocardial infarction (AMI) was diagnosed on the basis of the triad of chest pain, electrocardiogram changes, and elevated serum cardiac enzyme levels [15]. The criteria of stable angina pectoris was diagnosed on the basis of the symptom of exertional chest pain, angiographic evidence, and/or electrocardiogram changes, and normal serum cardiac enzyme levels. The criteria of unstable angina pectoris was diagnosed on the basis of criteria of AP and ischemic chest discomfort that increased or occurred at rest. The acute coronary syndrome (ACS) included AMI and unstable angina pectoris. The exclusion criteria included malignancies, pregnancy, end stage renal disease and severe liver or hematological diseases. These inclusion and exclusion criteria were met by 3365 continuously enrolled CAD patients. After excluding patients with loss of follow-up (*n* = 287) and missing data of TG (*n* = 17), 3061 patients were included in the data analysis. The study protocol was approved by ethics committee of West China Hospital, Sichuan University in accordance with the Declaration of Helsinki. All subjects provided written informed consent before enrolment.

### Baseline characteristics

Demographic data, medical history, cardiovascular risk factor, vital signs at admission, medication at discharge, and final diagnosis were obtained from the patients’ electronic medical records and reviewed by a trained study coordinator. Blood sample were collected at admission, and serum lipid including TG, liver and kidney function, blood glucose, etc. were analyzed in the department of Laboratory Medicine, West China hospital, accredited by the College of American Pathologists. TG level was measured by enzymatic colorimetric assay (Cobas 8000 C702 Chemistry autoanalyzer, Roche Diagnostics, Germany). Hypertension was defined as those with systolic blood pressure (SBP) ≥ 140 mmHg and/or diastolic blood pressure (DBP) ≥ 90 mmHg and/or those receiving antihypertensive medications. Diabetes mellitus (DM) was diagnosed in patients who had previously undergone dietary treatment for diabetes, had received additional oral antidiabetic or insulin medication or had a current fasting blood glucose level of ≥7.0 mmol/L or random blood glucose level ≥ 11.1 mmol/L. Patients received care according to the usual practice; treatment was not affected by participation in this study.

### Follow-up and end points

The follow-up period ended on January 2013. Follow-up information was collected through contact with patients’ physicians, patients or their family. All data were corroborated with the hospital records. The primary end point in this study was all-cause mortality and the secondary end point was cardiovascular death, as documented in the database. Death was considered cardiac when it was caused by acute MI, significant arrhythmias, or refractory heart failure. Sudden unexpected death occurring without another explanation was included as cardiovascular death.

### Statistical analyses

We conducted the post-hoc analysis on a retrospective basis. Baseline demographics and clinical characteristics were compared among patients categorized by the tertiles of admission TG level in three groups. Continuous variables are expressed as the mean ± standard deviation (SD), and categorical variables are reported as counts and percentages. Analysis of variance (ANOVA) and chi-squared tests were used to test for differences among groups for continuous and categorical variables, respectively. To determine the association between TG levels and all-cause mortality, Kaplan–Meier curves by tertiles of TG level were constructed and examined using the log-rank test for comparison. Cox proportional hazards models were used to evaluate the relationship of tertiles of TG level with all-cause and cardiovascular mortality, initially unadjusted and subsequently adjusting for several covariates: age, sex, medical history [pre-hypertension, pre-diabetes mellitus, pre-myocardial infarction, pre- percutaneous coronary intervention (pre-PCI), pre- cardiac artery bypass graft (pre-CABG)], admission examination [systolic blood pressure, heart rate, body mass index (BMI) and Global Registry of Acute Coronary Events (GRACE) score)], admission lab test (blood glucose, serum creatinine, LDL-C and HDL-C), and severity of CAD (left main artery, three vessel diseases and number of stents) [16–18]. Cox proportional hazards regression models were also used to investigate the independent effect of TG level on all-cause and cardiovascular mortality in overall CAD patients and subgroups. Two-sided *p* values of less than 0.05 indicated statistical significance. All analyses were performed with Stata MP (version 14.0).

## Results

A total of 3061 patients with CAD were included in the study. The average age was 64.4 ± 10.7 years, and men accounted for 79.4% of the patients. Serum TG levels were measured within 24 h after admission. The patients were divided into three groups according to the tertiles of TG levels. As shown by the baseline data distribution in Table 1, the clinical features showed differences among groups. Compared with lower TG level group, higher TG level group corresponded to higher TC and LDL-C levels, but lower HDL level, younger patient age, and better GRACE score.

**Table 1:** Baseline characteristics of the study population

Characteristics	Total	Tertile 1	Tertile 2	Tertile 3	*p* value
No. of patients	*n* = 3061	*n* = 1039	*n* = 1019	*n* = 1003	
Age, yrs	64.41 (10.68)	66.36 (10.21)	64.55 (10.33)	62.24 (11.09)	< 0.001
Gender, men, n (%)	2429 (79.4)	861 (82.9)	790 (77.5)	778 (77.6)	0.003
Medical history					
Pre-hypertension, n (%)	1684 (55.3)	568 (54.9)	581 (57.2)	535 (53.7)	0.270
Pre-diabetes mellitus, n (%)	665 (21.8)	187 (18.1)	259 (25.5)	219 (22.0)	< 0.001
Pre-myocardial infarction, n (%)	830 (27.1)	303 (29.2)	283 (27.8)	244 (24.3)	0.041
Pre-PCI, n(%)	365 (11.9)	143 (13.8)	114 (11.2)	108 (10.8)	0.076
Pre-CABG, n(%)	34 (1.1)	9 (0.9)	13 (1.3)	12 (1.2)	0.642
At admission					
Systolic blood pressure, mm Hg	130.72 (22.33)	129.09 (23.12)	131.21 (22.92)	131.91 (20.76)	0.013
Diastolic blood pressure, mm Hg	76.49 (12.57)	75.31 (12.76)	76.58 (12.38)	77.62 (12.46)	< 0.001
Heart rate, beats/min	74.34 (22.98)	74.88 (29.59)	74.30 (22.92)	73.82 (13.11)	0.584
Killip classification ≥ II, n (%)	345 (11.3)	136 (13.1)	121 (11.9)	88 (8.8)	0.007
GRACE score	92.74 (26.00)	96.44 (25.92)	93.32 (25.94)	88.30 (25.49)	< 0.001
BMI	24.40 (6.75)	23.55 (3.34)	24.72 (10.34)	24.94 (4.23)	< 0.001
Laboratory values					
Serum creatinine, μmol/L	93.90 (50.39)	89.46 (30.49)	96.19 (57.84)	96.19 (58.01)	0.002
Blood glucose, mmol/L	6.95 (3.13)	6.57 (2.75)	7.09 (3.27)	7.20 (3.294)	< 0.001
Total cholesterol, mmol/L	4.07 (1.12)	3.69 (0.94)	4.02 (1.00)	4.52 (1.25)	< 0.001
TG, mmol/L	1.75 (1.13)	0.91 (0.18)	1.48 (0.19)	2.89 (1.32)	< 0.001
LDL, mmol/L	2.46 (3.48)	2.14 (0.80)	2.41 (0.89)	2.83 (5.95)	< 0.001
HDL, mmol/L	1.15 (0.35)	1.26 (0.34)	1.15 (0.36)	1.03 (0.31)	< 0.001
Hemoglobin, g/L	134.39 (31.12)	131.54 (16.76)	133.48 (18.05)	138.28 (48.10)	< 0.001
White blood cell, n×10^9^/L	7.48 (4.94)	7.13 (3.00)	7.69 (7.06)	7.64 (3.78)	0.017
Severity of CAD					
Left main artery, n (%)	284 (9.3)	95 (9.1)	102 (10.0)	87 (8.7)	0.575
Three vessel diseases, n (%)	795 (26.0)	246 (23.7)	280 (27.5)	269 (26.8)	0.109
No. of stents	1.22 (1.22)	1.17 (1.19)	1.26 (1.21)	1.24 (1.27)	0.195
Diagnose					
ACS, n(%)	2174 (71.0)	715 (68.8)	715 (70.2)	744 (74.2)	0.022
AMI, n(%)	636 (20.8)	219 (21.1)	213 (20.9)	204 (20.3)	0.912
Discharge medications					
Aspirin, n (%)	2812 (91.9)	951 (91.5)	938 (92.1)	923 (92.0)	0.963
Clopidogrel, n (%)	2735 (89.3)	905 (87.1)	917 (90.0)	913 (91.0)	0.088
Statins, n (%)	2754 (90.0)	934 (89.9)	921 (90.4)	899 (89.6)	0.958
ACE inhibitors or ARBs, n (%)	1753 (57.3)	601 (57.8)	572 (56.1)	580 (57.8)	0.950
Beta-receptor blockers, n (%)	2028 (66.3)	634 (61.0)	671 (65.8)	723 (72.1)	< 0.001

Data are expressed as means ± SD or counts and percentages, as appropriate

*Abbreviations*: *BMI* body mass index, *PCI* percutaneous coronary intervention, *CABG* coronary artery bypass graft, *TG* triglyceride, *LDL-C* low-density lipoprotein cholesterol, *HDL-C* high-density lipoprotein cholesterol, *CAD* coronary artery disease, *ACE* angiotensin-converting enzyme, *ARBs* angiotensin-receptor blockers, *GRACE score* Global Registry of Acute Coronary Events score

**Fig. 1:**
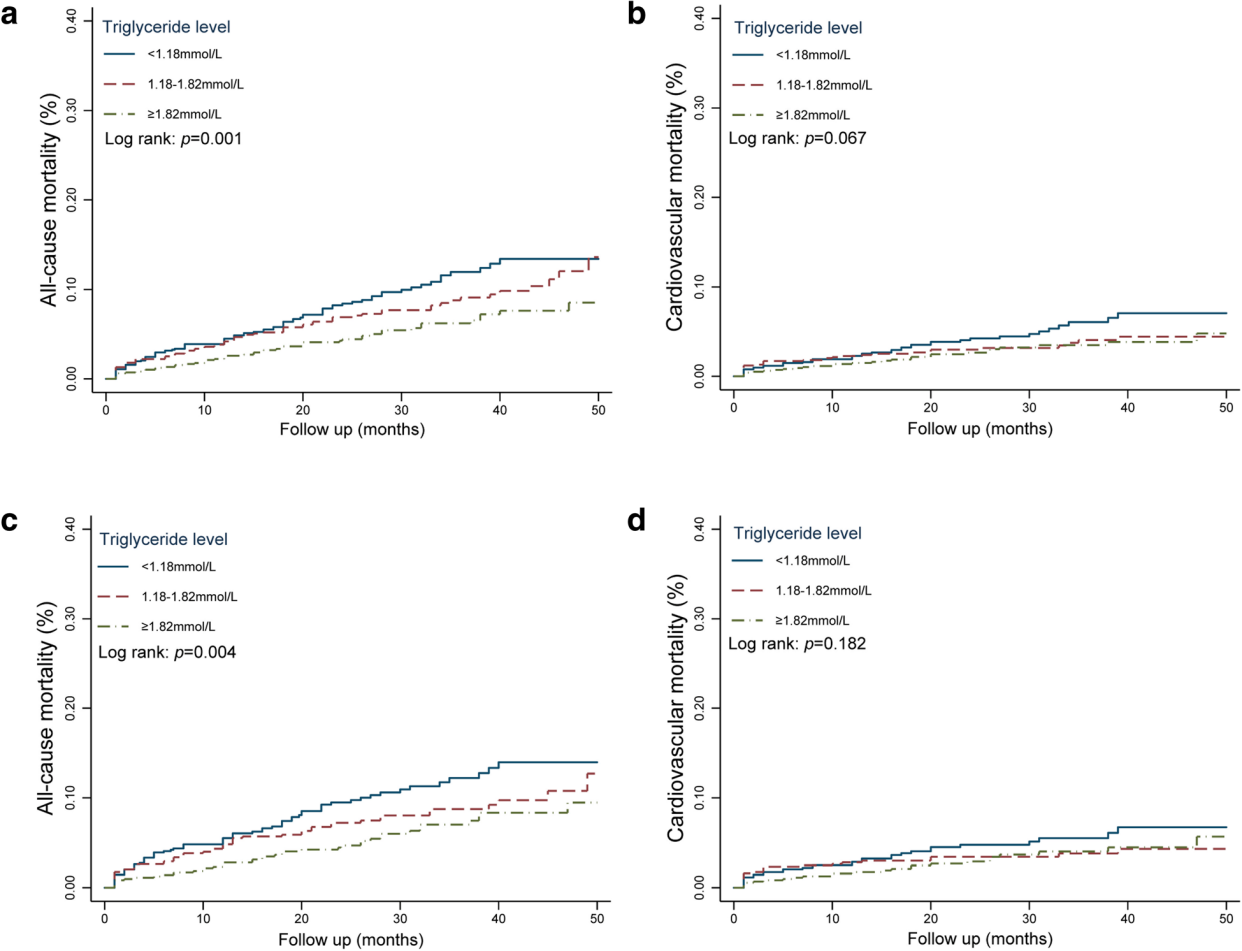
Kaplan–Meier survival curves for all-cause mortality and cardiovascular mortality patients with coronary artery disease according to triglyceride levels. Panel **a** and Panel **b** in total 3061 patients with CAD; Panel **c** and Panel **d** in 2174 patients with ACS

The 3061 patients were followed up for an average duration of 26.9 ± 13.6 months. Total of death events occurred in 258 cases (mortality rate: 8.4%) during the follow-up period, including 146 cases (cardiovascular mortality rate: 4.8%) of cardiac death. As shown by the cumulative survival curves of groups by tertiles of TG level, the risk of all-cause mortality exhibited an overall rising trend as TG levels decreased (mortality rate, Tertile 1 vs. Tertile 2 vs. Tertile 3, 10.3% vs. 8.6% vs. 6.3%, log rank test for overall *p* = 0.001) (Fig. 1, panel a). The risk analysis of cardiac mortality obtained results similar trend to those mentioned above (cardiovascular mortality rate, Tertile 1 vs. Tertile 2 vs. Tertile 3, 5.9% vs. 4.4% vs. 4.0%, log rank test for overall *p* = 0.067) (Fig. 1, panel b). Even after multifactorial regression, TG level were still inversely correlated with risk of all-cause mortality and cardiovascular mortality (Table 2). Furthermore, similar trends were detected in patients with ACS (Fig. 1, panel c and panel d; and Table 2).

**Table 2:** Relationship of tertiles of triglyceride level to all-cause mortality in coronary artery disease

	Tertile 1	Tertile 2	Tertile 3
Total patients			
All-cause mortality, HR (95% CI)			
Unadjusted	1.00	0.80 (0.59–1.09)	0.53 (0.38–0.75)
Adjusted^a^	1.00	0.75 (0.53–1.05)	0.49 (0.32–0.74)
Cardiovascular mortality, HR (95% CI)			
Unadjusted	1.00	0.69 (0.44–1.09)	0.60 (0.37–0.95)
Adjusted^a^	1.00	0.57 (0.35–0.93)	0.46 (0.27–0.81)
ACS patients			
All-cause mortality, HR (95% CI)			
Unadjusted	1.00	0.72 (0.51–1.04)	0.53 (0.36–1.04)
Adjusted^a^	1.00	0.66 (0.45–0.97)	0.53 (0.34–0.82)
Cardiovascular mortality, HR (95% CI)			
Unadjusted	1.00	0.68 (0.41–1.15)	0.65 (0.38–1.10)
Adjusted^a^	1.00	0.53 (0.31–0.94)	0.49 (0.27–0.89)

*Abbreviations*: *HR* hazard ratio, *ACS* acute coronary syndrome, *CI* confidence interval, *LDL-C* low-density lipoprotein cholesterol, *HDL-C* high-density lipoprotein cholesterol, *CAD* coronary artery disease, *BMI* body mass index, *GRACE score* Global Registry of Acute Coronary Events score.

^a^Risk factors adjustment included age, sex, medical history (pre-hypertension, pre-diabetes mellitus, pre-myocardial infarction, pre-PCI and pre-CABG), admission examination (systolic blood pressure, heart rate, BMI and GRACE score), admission lab test (blood glucose, serum creatinine, LDL-C and HDL-C), and severity of CAD (left main artery, three vessel diseases and number of stents)

Cox regression analysis showed an independent correlation between TG level and risk of all-cause mortality and cardiovascular mortality in total patients with CAD. Subgroup analysis found the similar results in patients with ACS and AMI, except the cardiac mortality in patients with stable angina pectoris (Table 3).

**Table 3:** Relationship of triglyceride level with all-cause mortality in coronary artery disease and subgroups^a^

	All-Cause Mortality		Cardiovascular Mortality	
	HR (95% CI)	*p*	HR (95% CI)	*p*
Total patients				
Unadjusted	0.74 (0.62–0.87)	< 0.001	0.77 (0.60–0.97)	0.028
Adjusted^b^	0.71 (0.58–0.86)	0.001	0.67 (0.51–0.89)	0.006
ACS				
Unadjusted	0.71 (0.60–0.88)	0.001	0.80 (0.61–1.04)	0.096
Adjusted^b^	0.72 (0.58–0.90)	0.003	0.69 (0.51–0.94)	0.018
AMI				
Unadjusted	0.63 (0.46–0.87)	0.005	0.53 (0.33–0.85)	0.008
Adjusted^b^	0.71 (0.50–1.01)	0.057	0.51 (0.30–0.86)	0.012
Stable angina pectoris				
Unadjusted	0.74 (0.52–1.06)	0.099	0.62 (0.36–1.06)	0.086
Adjusted^b^	0.61 (0.39–0.95)	0.028	0.59 (0.24–1.16)	0.127

*Abbreviations*: *HR* hazard ratio, *CI* confidence interval, *ACS* acute coronary syndrome, *AMI* acute myocardial infarction, *LDL-C* low-density lipoprotein cholesterol, *HDL-C* high-density lipoprotein cholesterol, *CAD* coronary artery disease, *BMI* body mass index, *GRACE score* Global Registry of Acute Coronary Events score

^a^Triglyceride levels was grouped by the tertiles; ^b^Risk factors adjustment included age, sex, medical history (pre-hypertension, pre-diabetes mellitus, pre-myocardial infarction, pre-PCI and pre-CABG), admission examination (systolic blood pressure, heart rate, BMI and GRACE score), admission lab test (blood glucose, serum creatinine, LDL-C and HDL-C), and severity of CAD (left main artery, three vessel diseases and number of stents)

## Discussions

This study found an inverse association between TG levels and mortality risk in patients with CAD, which suggests that the “TG paradox” may exist in CAD patients.

The association between TG and ASCVD risk has not been less certain than that for LDL-C for a long time. Some studies suggested that TG has no association with CAD development or cardiovascular events [7, 19]. Some clinical trials did not find any direct improvement in primary outcomes in response to fibrate treatment [20]; meanwhile, no further decrease was found in cardiovascular events when comparing combination lipid therapy (statins and fibrates) with statin therapy alone [21]. Therefore, in the recommendation guidelines, HTG treatment is the second recommendation after LDL-C control [22]. A few recent studies found that low TG is associated with high mortality risk and poor prognosis in stroke; these studies then proposed the concept of a “TG paradox” [11, 14]. Similar to stroke, CAD is an atherosclerotic disease, which prompts the following question: does the TG paradox also exist for cardiovascular disease? Two recent small-sample studies have provided some clues. Cheng et al. found that fasting the TG levels measured within 24 h of admission were inversely associated with hospital mortality and long-term prognosis such as target vessel revascularization and overall major adverse cardiac events, as indicated by an analysis of 247 ST elevation myocardial infarction (STEMI) patients undergoing primary PCI [23, 24]. Khawaja et al. observed a higher 3-year mortality risk in patients with lower fasting TG levels measured within 24 h of admission, as indicated by an analysis of 517 non-ST elevation myocardial infarction (NSTEMI) patients [24]. In the present study, our result is similar to the findings reported in the above two studies and it indicates that the “TG paradox” is present in the CAD cohort of our study.

Currently, the mechanism underlying the “TG paradox” is still unclear and may be related to the following factors. First, low TG levels may reflect the overall poor nutritional status of patients. Epidemiological and clinical controlled studies showed that TG levels are significantly influenced by the state of body weight and the distribution of body fat. Data from National Health and Nutrition Examination Surveys demonstrated a significant correlation between BMI and TG levels [25]. The Framingham Heart Study revealed that TG levels are strongly associated with abdominal subcutaneous fat and visceral fat distribution [26]. The “obesity paradox” is present in particular chronic wasting diseases, such as CAD, heart failure, and chronic kidney disease, wherein BMI levels are inversely associated with mortality risk [27–29]. Thus, the TG level, which is significantly affected by BMI, may also present a similar paradox. Second, heparin use stimulated the release of lipoprotein lipase from endothelial cells, which can reduce the TG levels in blood circulation [30, 31]. Meanwhile, the greater sympathetic activity in acute phase of myocardial injury directly provokes the synthesis rate and activation of lipoprotein lipase, which breaks the TG from circulating state down to glycerol and fatty acids [31–34] It was previously reported that TG levels significantly decrease in ACS patients compared with stable angina pectoris patients within 24 h of onset and the low TG level were associated with the recurrent ischemia and the higher 30-days mortality in ACS patients [35–37]. Third, other basic research reported that HTG may have a potential protective role in vascular lesions. HTG can provide protective effects against fatty acid-induced lip toxicity [38]; additionally, blood lipid levels that are too low are detrimental to maintaining a stable state of cell membranes [39]. Finally, our study included a limited number of adjustment factors for the regression equation, and some unknown risk factors associated with TG might be overlooked, thus exaggerating the role of TG in the secondary prevention of CAD. Therefore, due to lack of large sample prospective study that is specifically designed to address the mechanism underlying this issue, but it is still inconclusive whether TG directly affects the prognosis of CAD patients or just plays a role as an indirect factor. These issues need to be further investigated.

This study has a few limitations. First, the study was a single-center observational study. Despite the multi-factor adjustment, it was difficult to completely avoid selection bias and the remaining confounding factors. Second, observational studies do not positively interfere with any diagnosis and treatment of patients; thus, a random TG measurement, other than the fasting TG, is used for emergency patients. In this study, the fasting TG level was collected from most elective coronary angiography patients (mainly with stable angina pectoris or unstable angina pectoris), while a random TG level was used for most emergency PCI patients (mainly with AMI). Therefore, the TG levels would be more susceptible to the effects of meal and thus increase in high-risk patients. However, in the current study, the percentages of AMI patients were similar in the various TG groups, suggesting that our results were not significantly affected by meals. The last, our analysis included only the measurement of laboratory and heart echocardiography completed within one week after admission of the patients to the hospital, which were not conducted during stable phase.

## Conclusion

This study found an inverse association between TG levels and mortality risk in CAD patients, which suggests that the “TG paradox” may exist in CAD patients.

## Abbreviations

ACS: Acute coronary syndrome
AMI: Acute myocardial infarction
ANOVA: Analysis of variance
ASCVD: Atherosclerotic cardiovascular disease
BMI: Body mass index
CAD: Coronary artery disease
DBP: Diastolic blood pressure
DM: Diabetes mellitus
ESRD: End stage renal disease
HDL-C: High-density lipoprotein cholesterol
HTG: Hypertriglyceridemia
LDL-C: Low-density lipoprotein cholesterol
MACE: Major adverse cardiac events
NSTEMI: Non-ST elevation myocardial infarction
SBP: Systolic blood pressure
SD: Standard deviation
STEMI: ST elevation myocardial infarction
TC: Cholesterol
TG: Triglyceride
